# Supplementary material for: Biological Effects of Human Exposure to Environmental Cadmium
Source: Biomolecules. 2022 Dec 24;13(1):36. doi: 10.3390/biom13010036 (PMC9855641; doi:10.3390/biom13010036)
Supplement: Supplementary file 1 [file biomolecules-13-00036-s001.zip › biomolecules-2019153-supplementary.pdf]

# Biological Effects of Human Exposure to Environmental Cadmium

**Massimiliano Peana [1],* Alessio Pelucelli [1],* Christos T. Chasapis [2], Spyros P. Perlepes [3], Vlasoula Bekiari[4], Serenella Medici [1], and Maria Antonietta Zoroddu[1]**

1 Department of Chemical, Physical, Mathematical and Natural Sciences, University of Sassari, 07100 Sassari, Italy; peana@uniss.it (M.P.), alessiopelucelli@gmail.com (A.P.), sere@uniss.it (S.M.), zoroddu@uniss.it (M.A.Z.)
2 Institute of Chemical Biology, National Hellenic Research Foundation, 11635 Athens, Greece; cchasapis@eie.gr
3 Department of Chemistry, University of Patras, 26500 Patras, Greece; perlepes@upatras.gr
4 School of Agricultural Science, University of Patras, 30200 Messolonghi, Greece; bbekiari@upatras.gr

* Correspondence: peana@uniss.it (M.P.), alessiopelucelli@gmail.com (A.P.)

## Supplementary Material

**Table S1.** Percentage distribution of geometries for $Cd^{2+}$ complexed with proteins according to RCSB PDB database

| Geometry | N° entry | % |
|---|---|---|
| octahedron | 60 | 12,7% |
| square pyramid with a vacancy (equatorial) | 68 | 14,3% |
| square pyramid | 31 | 6,5% |
| square plane | 15 | 3,2% |
| square plane with a vacancy (regular) | 30 | 6,3% |
| tetrahedron with vacancy | 51 | 10,8% |
| tetrahedron | 24 | 5,1% |
| trigonal plane with a vacancy | 85 | 17,9% |
| trigonal bipyramid with a vacancy (axial) | 21 | 4,4% |
| trigonal bipyramid with a vacancy (equatorial) | 17 | 3,6% |
| trigonal bipyramid | 4 | 0,8% |
| pentagonal bipyramid with a vacancy (equatorial) | 33 | 7,0% |
| pentagonal bipyramid | 16 | 3,4% |
| others | 19 | 4,0% |

**Table S2.** Percentage distribution of metal ions substituted by $Cd^{2+}$ according to the RCSB PDB database

| Metals | % of Cd substitution in Metal-protein | n° of Cd substitution in Metal-protein | % of Cd substitution in Metal-proteins |
|---|---|---|---|
| Zn | 4,1% | 1245 | 29,7% |
| Mg | 2,1% | 623 | 14,0% |
| Ca | 2,2% | 590 | 14,1% |
| Co | 1,6% | 417 | 10,0% |
| Mn | 1,1% | 368 | 8,8% |
| Fe | 3,2% | 309 | 7,4% |
| Na | 1,1% | 286 | 6,8% |
| Cu | 1,6% | 167 | 4,0% |
| Ni | 0,9% | 134 | 3,2% |
| K | 0,5% | 50 | 1,2% |

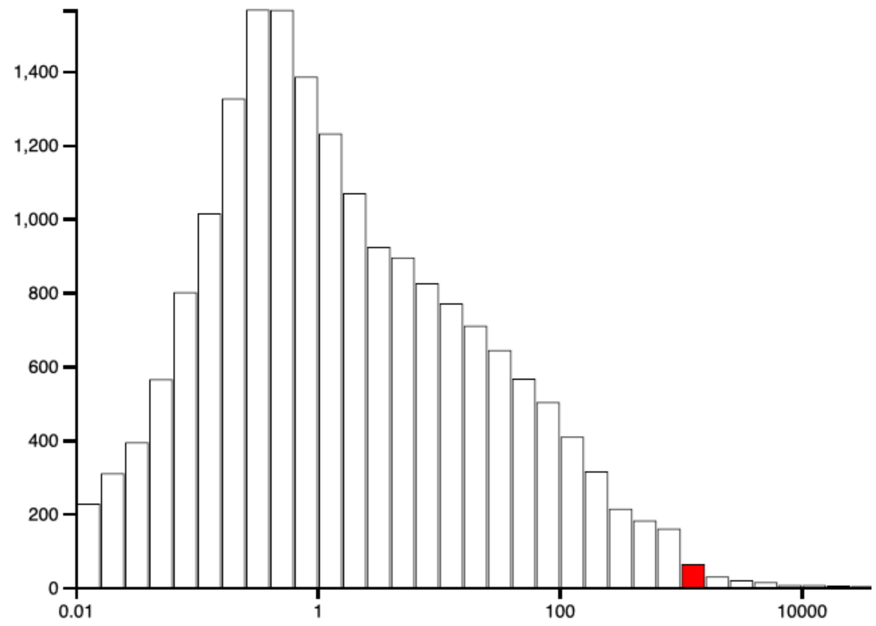


**Figure S1.** Protein abundance histogram of human proteins. In red the abundance of Heat Shock protein HSP 90-beta.
